# MicroRNAs in Papillary Thyroid Cancer: What Is New in Diagnosis and Treatment

**DOI:** 10.3389/fonc.2021.755097

**Published:** 2022-02-03

**Authors:** Maria Papaioannou, Angeliki G. Chorti, Anthoula Chatzikyriakidou, Kleanthis Giannoulis, Sohail Bakkar, Theodosios S. Papavramidis

**Affiliations:** ^1^ Laboratory of Biological Chemistry, School of Medicine, Faculty of Health Sciences, Aristotle University of Thessaloniki, Thessaloniki, Greece; ^2^ 1st Propedeutic Department of Surgery, American Hellenic Educational Progressive Association (AHEPA) University Hospital, Aristotle University of Thessaloniki, Thessaloniki, Greece; ^3^ Laboratory of Medical Biology, School of Medicine, Faculty of Health Science, Aristotle University of Thessaloniki, Thessaloniki, Greece; ^4^ Department of Surgery, Faculty of Medicine, The Hashemite University, Zarqa, Jordan

**Keywords:** microRNAs (miRNAs), papillary thyroid cancer, thyroid cancer, microRNA thyroid, diagnostic test

## Abstract

**Introduction:**

Papillary thyroid cancer (PTC) accounts for up to 80% of thyroid malignancies. New diagnostic and therapeutic options are suggested including innovative molecular methods. MicroRNAs (miRNAs) are nonprotein coding single-stranded RNAs that regulate many cell processes. The aim of the present study is to review the deregulated miRNAs associated with PTCs.

**Methods:**

A bibliographic research was conducted, resulting in 272 articles referred to miRNAs and PTC. Regarding our exclusion criteria, 183 articles were finally included in our review.

**Results:**

A remarkably large number of miRNAs have been found to be deregulated during PTC manifestation in the literature. The deregulated miRNAs are detected in tissue samples, serum/plasma, and FNA samples of patients with PTC. These miRNAs are related to several molecular pathways, involving genes and proteins responsible for important biological processes. MiRNA deregulation is associated with tumor aggressiveness, including larger tumor size, multifocality, extrathyroidal extension, lymphovascular invasion, lymph node and distant metastasis, and advanced tumor node metastasis stage.

**Conclusion:**

MiRNAs are proposed as new diagnostic and therapeutic tools regarding PTC. They could be essential biomarkers for PTC diagnosis applied in serum and FNA samples, while their contribution to prognosis is of great importance.

## Introduction

Thyroid cancer is the fifth most frequent cancer in women and gains the forefront among endocrine gland malignancies. Papillary thyroid cancer (PTC) is the most common type of thyroid cancer, and its global incidence has increased rapidly in the last decades (1). The diagnostic and therapeutic process for PTC is determined by Thyroid Associations Guidelines, suggesting ultrasound and Fine-Needle Aspiration (FNA) as diagnostic tools until now and surgery as the treatment option (2). Nevertheless, FNA cytology may be inconclusive. Furthermore, guidelines have proposed specific clinicopathological features, such as age, gender, cancer subtype, tumor size >2 cm, extrathyroidal extension, multicentricity, and BRAF V600E mutation, to be indicative of recurrence and poorer prognosis (2). Novel molecular biomarkers should be discovered and applied as useful tools for the diagnostic and therapeutic management of PTC, so that the lesions are better characterized regarding their malignancy and aggressiveness.

MicroRNAs (MiRNAs) are endogenous, nonprotein coding single-stranded RNAs containing between 19 and 24 nucleotides. These molecules have been found in the genome of the majority of complex organisms (3). The expression of miRNAs is initiated in cell nuclei by the transcription of miRNA genes. The first product, named pri-miRNA, is cleaved by the Drosha DGCR8 (DiGeorge critical region 8) complex to become pre-miRNA. Pre-miRNA is then exported to the cytoplasm by exportin-5 and matures to miRNA-duplex by the Dicer enzyme. One strand is the guide strand, which enters the RNA-induced silencing complex (RISC), and the other one is the passenger strand, which is degraded. Mature miRNA binds to the 3’-UTR end of mRNA, and the stability or translation of mRNA is determined by the complementarity between the two molecules (4). Due to the range of complementary binding between miRNA and mRNA, miRNA can bind with several mRNAs and to regulate a wide variety of protein-coding gene transcripts. MiRNA plays an essential role in post-transcriptional processes, being involved in the regulation of a large number of proteins responsible for many cell functions (5). Taking into account the binding facility and regulatory effects of miRNAs, their deregulation, due to their abnormal expression, may be involved in many biological processes, whose deregulation may be associated with cancer development (4). MiRNA database counts 38,589 entries until now, and these molecules and their regulatory networks are being explored as new diagnostic and therapeutic targets for many diseases (6, 7).

The aim of the present systematic review is to examine the most important deregulated miRNAs in PTCs, defined as the most frequently detected molecules in the majority of international genetic studies.

## Methods

**Figure 1:**
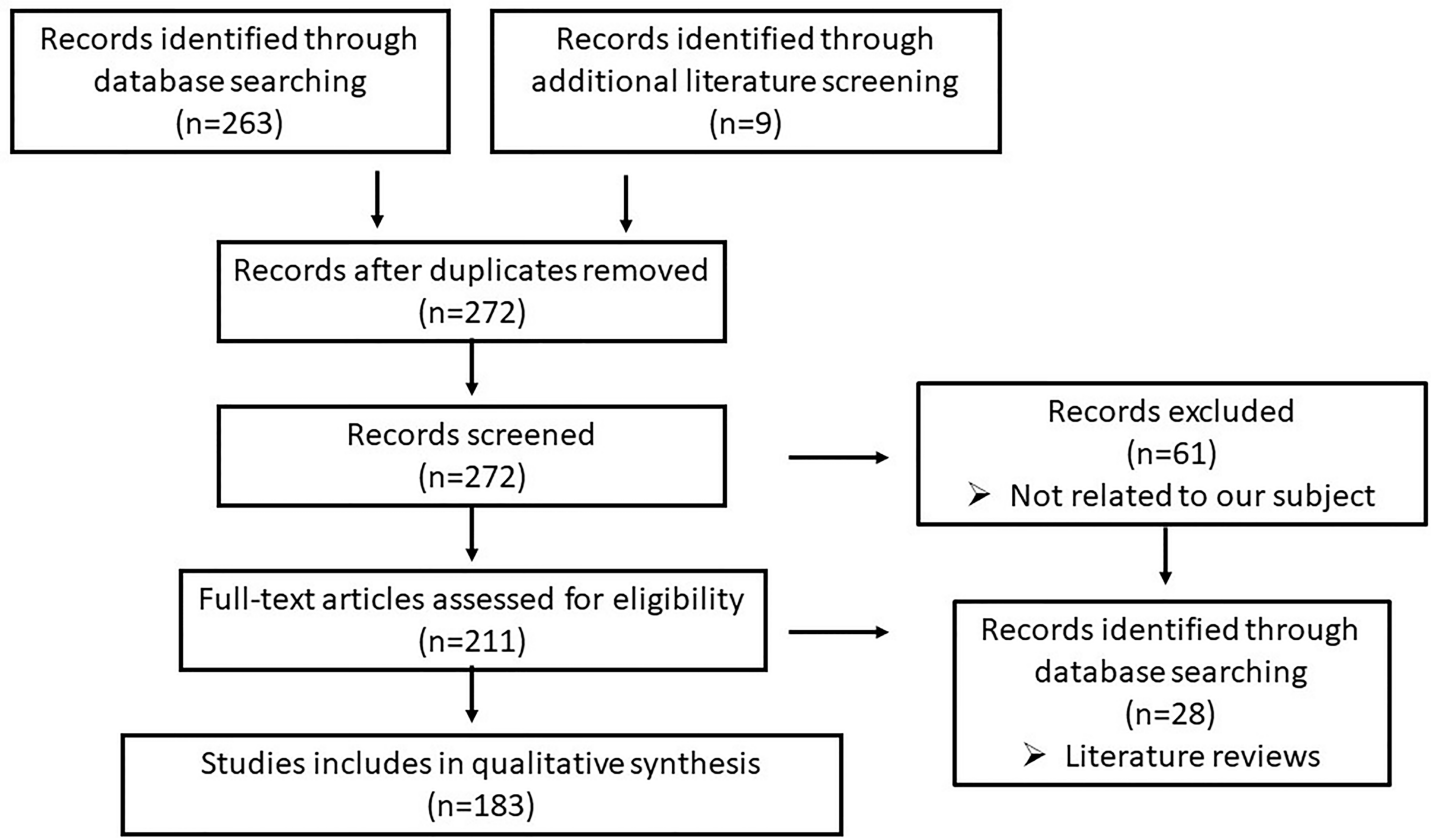
Flow diagram.

A bibliographic research was conducted using PubMed, Scopus, and Embase from 2010 until January 2021. The search terms employed were “microRNAs” OR “miRNAs” AND ‘‘ papillary thyroid cancer” OR “papillary thyroid carcinoma”. There were 263 articles found in these databases and 9 in the literature, from which only 211 articles were associated with our subject. Only well-conducted genetic studies aiming to explore the association between miRNAs and their diagnostic and therapeutic application in PTC were included in our systematic review, so 28 articles were excluded as they were literature reviews. Finally, our article database included 183 articles (**Figure 1**).

## Results

### MicroRNAs Upregulated in Thyroid Specimen

A large number of upregulated miRNAs are detected in PTC specimens as summarized several studies internationally (4, 8, 9).

MiRNA 146a and 146b are widely the most well-studied deregulated miRNA in tumorous thyroid tissues. They are upregulated in thyroid specimens compared with normal tissue and benign thyroid pathology, as summarized in the Chou et al. review (10). The overexpression of mir146a was found to be due to the decreased ubiquitin-proteasome mediated degradation of HIFα, owning to increased activity of LSD1. Mir-146a inhibited the expression of GABPA and in this way contributed to the inhibition of apoptosis and stimulation of PTC cell malignancy (11). MiR146b targeted molecular pathways such as the MAPK/ERK pathway and TGF-β and was found to be involved in several cellular functions such as actin cytoskeleton formation affecting thyroid cell migration and invasion (10, 12). In particular, miR-146b targets the IL-1 receptor-associated kinase 1 (IRAK1), in which its inhibition is correlated with increased PTC cell proliferation, tumor invasiveness, and aggressiveness, probably due to the deregulation of the E-cadherin-mediated EMT (13, 14). MiR-146b-5p plays a significant role in PTC development, affecting cell proliferation and invasion, which is enhanced during the TGF-β1-induced EMT (epithelial–mesenchymal transition) signaling pathway (15). Another negative regulator of the Wnt/β-catenin pathway involved in the EMT is ZNRF3, which is also downregulated by miR-146b-5p (10, 16). Moreover, MiR146b-5p targets both the gene expression of *RARβ* and *CCDC6* and promotes tumor development (17, 18). Another family member is MiR146b-3p, which is highly expressed in metastatic cell lines and enhances cell invasion and metastasis by suppressing the NF2 gene expression (19). The reduced expression of the *THRβ* is mediated by miR-146a (20). The increased expression of MiR146b can be due to DNA hypomethylation and therefore increased expression. The upregulation of MiR146b can be a sensitive (91–96%) and specific (96–97%) marker for the discretion of benign and malignant thyroid lesions and a prognostic marker for recurrence (21–23). Moreover, the expression of the miR146 family is associated with the tumor stage according to TNM, tumor aggressiveness, classical tall cell variant of PTC (in particular miR-146b-5p), and increased lymph node metastasis risk (14, 20, 22, 24–26). Interestingly the expression of mir-146b-5p was higher in PTC specimens without marked lymphocytic infiltration, compared to specimens from lymphocytic thyroiditis, indicating that mir-146b-5p could contribute to the inhibition of NKG2D and to the escape of the immune response (27). MiR146a overexpression in tissue was correlated with female gender, greater size, central lymph node metastasis, multifocality, extrathyroidal extension, and advanced tumor stage in TNM according to statistical analysis results of a cohort study by Sun et al. (28).

MiRNA 221 and 222 were also found frequently upregulated in PTC specimens due to the interaction of the high-mobility group box 1 protein (HMGB1) with the receptor for advanced glycation end-products (RAGE). The activation of the HMGB1/RAGE pathway beside the contribution to the chronic inflammation and the inhibition of the phosphatase and tensin homolog (PTEN), a regulator of the cell, induces the expression of miR-221 and -222 and contributes to PTC development (29, 30). MiR-221 binds to the TIMP3 mRNA. As result of the induced *TIMP3* gene suppression, the cell proliferation and PTC growth and aggressiveness are increased (31). Moreover, a negative correlation was found among the expression of miR-221, -222, -146b, and p27(kip1) mRNA levels in PTC cells (32). Upregulation of miR-221 possibly through interleukin 17 and miR-222 is correlated with the advanced TNM stage, capsular invasion, extrathyroidal extension, and lymph node metastasis, a fact that is also included in American Thyroid Association risk factors guidelines (22, 33, 34). A cohort study conducted by Dai et al. showed that both miR-221 and -222 are predictive biomarkers of PTC recurrence. However, only miR-221 was the independent factor (35). The downregulation of the *THRβ* gene was also inversely correlated with the overexpression of miR-221 and was associated with the aggressiveness of PTC (20). MiR-222 directly targets the 3′-UTR of *PPP2R2A*, which expresses the Protein Phosphatase 2 Regulatory Subunit B Alpha (PPP2R2A), a tumor suppressor, altering the Akt signaling pathway, and therefore enhancing the tumor metastasis in nude mice (36, 37). Suresh et al. imply that racial disparity plays a role in miR-221 overexpression, However, there is no race-specific difference in miR-222 upregulation in PTC patients (38).

MiR-595 expression was found to be upregulated in PTC cells resulting to the downregulation of the sex-determining region Y-box 17 (Sox17) and consequently the overexpression of the inflammatory cytokine interleukin (IL22), which promoted cell migration (39). Increased cell proliferation, migration, and *in vivo* transplantation were also seen due to the miR-1270 binding to *SCAI* and its inverse regulation (40). Interestingly, the expression of MiR-21 was found to be increased in PTC cell lines under hypoxic conditions and promoted angiogenesis through direct targeting and inhibition of *TGFB1* and *COL4A1* gene expression. Therefore, the increased endothelial tube formation and angiogenesis could be associated with the increased PTC recurrence (41, 42). Another possible oncogenic pathway for miR-21 action implied *PDCD4* downregulation and enhancement of cell proliferation and invasion (43). According to Rosignolo et al., in addition to the correlation of the overexpression of miR-21 with increased risk of recurrence, there is a statistically significant correlation with tall cell variant in PTC specimens (22). MiR-183 is another oncogene described, which, by inhibiting the expression of the *PDCD4* gene, enhanced the PTC progression and inhibited apoptosis (44). MiR-182 was also shown to promote tumor growth and invasion by inhibiting the *CHL1* gene expression (45). Overexpression of miR-92a-3p was found to be associated with PTC nodal metastases, whereas a negative correlation between the expression of the VHL and miR-92a was observed in PTC specimens with vascular invasion (46). Fang et al. found that miR-625-3p overexpression promoted the expression of the astrocyte elevated gene 1 (*AEG1)* and induced the activation of Wnt/β-catenin and JNK signaling cascade, which promoted the proliferation, migration, and invasion of thyroid cancer cells (47). The oncogene function of miR-96 was shown to be mediated by suppressing the expression of FOXO1, affecting the signaling pathway of the Akt/FOXO1/Bim axis, and therefore mediated the proliferation and the survival of PTC cells (48). The reduced expression of the *THRβ* gene was also found to be negatively correlated with the increased expression of miR181a in PTC specimens (20). CYLD, a member of the Wnt/β catenin pathway and a negative regulator of NF-κB, was found to be underregulated by MiR-181b, which was overexpressed in PTC specimens (49). Therefore, both miR-181a and miR-181b are suggested to be possible therapeutic targets in PTC therapy.

MiR-155 is referred to as a biomarker for the discrimination between benign and malignant thyroid lesions and for greater size, extrathyroid extension, central lymph node metastasis, advanced TNM stage, and poor prognosis (50). Upregulated miR-34a and -424 are essential markers for tumor aggressiveness, as indicated by bioinformatics analysis of data derived from the Cancer Genome Atlas (51). The has-miR-200a-5p as an overexpressed molecule is a sensitive biomarker and could be combined with immunohistochemical markers such as TPO, CD56, Galectin 3, MC, CK19, and BRAF both to detect and to distinguish between PTC and benign thyroid tumor with papillary hyperplasia (52).

The Let-7c microRNA family is known to have an important role in PTC tumorigenesis mainly by reducing RAS levels and acting as a tumor suppressor gene, as summarized in the Perdas et al. review (53). However, let-7b and let-7c were found to be overexpressed in PTC samples.

Other microRNAs that are found in international studies to be upregulated are miR- 196a-5p, -31, -187, -551-3p, -99b, -340, -954, -18a, -506, -578, -381, -3613, and -346 (4, 24, 38, 54, 55) (**Table 1**). The effects of upregulated miRNA in papillary thyroid cancer cell lines and tissue are summarized in **Supplementary Table 1**.

**Table 1:** Upregulated miRNA in papillary thyroid cancer.

MiRNA	Tissue	Plasma/Serum
146a	+	+
146a-5p	+	+
146b	+	+
146b-5p	+	+
221	+	+
221-3p		+
222	+	+
222-3p		+
595	+	
1270	+	
21	+	+
183	+	
182	+	
92a	+	
625-3p	+	
96	+	
181	+	+
Let-7b	+	
Let-7c	+	
Let-7e		+
155	+	+
34a	+	
424	+	
200a-5p	+	
196a-5p	+	
31	+	+
187	+	
551-3p	+	
99b	+	
340	+	
954	+	
18a	+	
506	+	
578	+	
381	+	
3613	+	
346	+	
451a		+
30a-5p		+
106a		+
Let-7e		+
191-5p		+
93-5p		+
Let-7b		+
Let-7b-5p		+
24-3p		+
103a-3p		+
28-3p		+
423-5p		+
22	+	+
25-3p		+
190		+
95		+
10a-5p		+
598-5p		+
3161		+
4644		+
6516-5p		+
1283		+
5189-3p		+
4433a-5p		+
485-3p		+
151-5p		+

### MicroRNAs Downregulated in Thyroid Specimen

A notable group of downregulated miRNAs, influencing genes’ expression and cellular processes, has been also found in both in PTC cell lines and specimens. They regulate cell cycle and proliferation, migration, and invasion, such as miR-7, which was found to be downregulated in PTC samples. However, upon the overexpression of miR-7, the expression of the oncogene *CKS2* (cyclin-dependent kinase regulatory subunit 2) is suppressed, affecting downstream cell cycle regulation and resulting to cell cycle arrest in the G0/G1 phase (56). Interestingly, the cotreatment of PTC cells with mir-7-5p and chemotherapeutics loaded on cubosomes statistically significantly inhibited the proliferation and spheroid formation (57). Also, the overexpression of miR-791, found to be underexpressed in PTC specimens, induced cell cycle in the G0/G1 phase by inhibiting the expression of cyclin D1, CDK6, and CDK4 (58). The downregulation of miR-144 expression in PTC is associated with a larger tumor size. MiR-144 inhibited the cell proliferation by targeting the WW domain−containing transcription regulator 1 (WWTR1) (59). Moreover, miR-144 targeted the transcription factor E2F8 and induced cell cycle arrest in G1-phase arrest by downregulating Cyclin D1 (60). Mir144-3p was found to be suppressed by BAG5, and therefore the expression of fibronectin 1 (FN1) was maintained to be increased, promoting the invasion in PTC cells (61). The direct interaction of miR-144-3p with FN1 was also found to mediate the oncogenic function of SphK1 in PTC cells (62).

The downregulation of miR-1266 in PTC could be responsible for the overexpression of FGFR2 (63). MiR-335 acted also as a tumor suppressor by targeting the zinc finger E-box binding homeobox 2 (ZEB2) (64). Another form, the miR-335-5p, could target intercellular adhesion molecule 1 (ICAM-1) and accelerates cell migration and invasion (65). Through the Akt/mTOR pathway, miR-718 targeted phosphoinositide-dependent protein kinase 1 (PDPK1) and inhibited cell glucose metabolism and PTC progression (66). MiR-148a increased Bax expression and caspase−3/9 levels and enhanced apoptosis. The inhibition of lymphatic metastasis of PTC was due to suppressed phosphorylation of STAT3 and the inhibition of PI3K/Akt signaling. Inhibition of CDK8 expression was found to be another mechanism of miR-148a-mediated inhibition of PTC cell growth, migration, and invasiveness (67, 68). One of the most well-studied oncogenes for thyroid malignancies is *BRAF*, in which its 3’-untranslated region was found to be directly targeted by miR-9-5p, and therefore was negatively regulated in papillary cell lines (69). MiR-449 and miR-202-3p was found to be negative regulators of PTC through inhibiting the Wnt/β-catenin signaling pathway (70, 71). MiR-126 was found to be underexpressed both in PTC specimens and cells, whereas its functional role lies on the inhibition of VEGF-A-mediated angiogenesis (72). Moreover, miR-126 was found to regulate the Wnt/β-catenin signaling pathway *via* regulating the low-density lipoprotein receptor−related protein 6 (LRP6). The aberrant expression of miR-126 was found to be related to lymph node metastasis and advanced TNM stage (73). VEGF was found to be inhibited by miR-622 too. In PTC samples, the reduced expression of miR-622 was found to correlate with lymph node metastasis and advanced TNM stage (74). Also, the underexpression of miR-150 in PTC samples was found to be negatively correlated with TNM stage and lymph node metastasis. Mir-150 directly targeted the endogenous Rho−associated protein kinase 1 (ROCK1) (75). MiR-205 is another tumor suppressive molecule that targeted the action of both YAP1 and VEGFA and inhibited cell proliferation, cell cycle progression, and angiogenesis (76, 77).

MiR-204-5p and miR-7-2 were also described as tumor suppressors. Their underexpression in the PTC specimen indicated their possible role as tumor stage biomarkers. Mir-204-5p was found to be capable of inhibiting cancer progression by regulating the expression of TNRRSF12A, associated with angiogenesis, and IGFBP5, associated with cell cycle progression and proliferation (14, 78). Furthermore, the downregulation of miR-204 was found to be strongly related to follicular and tall cell variant type, extrathyroidal extension and metastasis of PTC, and the presence of BRAF-V600E mutation (22, 78, 79). Decreased Dicer gene expression in malignant tissues correlated greatly with aggressive features: extrathyroidal extension, angiolymphatic invasion, multifocality, lymph node and distant metastasis, and recurrence. The occurrence of BRAF-V600E mutation and the aggressive characteristics of PTC were found to be related with the decreased expression of DICER-mRNA (80). However, these results are controversial since Penha et al. found that DICER1 mRNA was overexpressed in 70% of the PTC samples. However, Dicer1 protein levels were downregulated and affected PTC proliferation and differentiation (81). MiR-451a, one of the most reported miRs, was found to be underexpressed in PTC tissue and associated with aggressive clinicopathological features, tall cell variant of PTC, extrathyroidal extension, and advanced tumor stage. Mir-451a targeted the expression of MIF, c-MYC, and AKT1 and thereby inhibited the Akt/mTOR signaling (82). *Via* the Akt pathway and by targeting the expression of *ARFGEF1* and *IRS2* genes, respectively, miR-215 and miR-766 detected in lower levels promoted lymph node metastasis, while miR-431 was suppressed in lymph node metastasis, accounting for an important biomarker for metastatic disease, regulating cytoskeleton formation by E-cadherin and Vimentin, and inhibiting the Hedgehog pathway (83–85). MiR-486-5p underexpression, KIAA1199 overexpression, and EMT formation were described to be connected to lymph node metastasis, TNM stage, recurrence, and survival (86, 87). MiR-486-5p targets the *FBN1* gene (88). MiR-940, -16, -15a, -126, and -199a-3p, miR-26a-5p, and miR-564 were found to be negatively regulated in PTC specimens and cell lines and were associated with bilateral tumor, multicentricity, extrathyroidal extension, cervical lymph node metastasis, distant metastasis, advanced TNM stage, and recurrence (89–93). A genetic study applying small RNA deep sequencing demonstrated that miR-30c-2-3p, -876-5p, -138-1-3p and -138-5p, -139-3p and -139-5p, -504, -152, -873-5p, and -199b-5p were downregulated in PTC specimens and mainly underexpressed in PTC with lymph node metastasis, concluding that these may be useful biomarkers for metastatic cancer (94). The induced overexpression of miR-139 in PTC cell lines inhibited the expression of FN1 (95). Furthermore, miR- 152 and -20b (by impairing the MAPK/ERK signaling pathway) were found to be related to more aggressive types of papillary thyroid cancer, advanced TNM stage, and lymph node metastasis (51, 96). Mir-326 was found to be downregulated in PTC specimens and cells, whereas there was a significant correlation with tumor stage and metastatic properties. Mir-326 could inhibit the progression of PTC by impairing Ki-67, N-cadherin, MAPK1, and ERBB4 (97). Through the MAPK signaling pathway, miR-TG participates in PTC progression. Interestingly, miR-TG was found to be encoded within the thyroglobulin (TG) gene (98). MiR-369-3p was detected as negatively regulated in classical, follicular, and tall cell variant of PTC, whereas the expression of the *TSPAN13* gene was enhanced (99). Another poorly expressed miR both in PTC tissue and cells was miR-448, which reduced expression was correlated with lymph node metastasis, and TNM stage (100). Mechanistically, the expression of the *miR-448* gene is inhibited by the binding of KDM5B, a specific lysine demethylase, resulting to the overexpression of TGIF1.

The Let miRNAs family, as already mentioned, was found to be involved in PTC development. Besides the upregulated family members, let-7f, -7d, and -7g were found to be downregulated in PTC samples, whereas let-7f suppressed the MAPK/ERK signaling pathway (53). Another family member, Let-7a, was found to negatively regulate the expression of *lin28* and *AKT2* genes and *via* the c-myc pathway is associated with TNM stage, lymph node metastasis, and recurrence (36, 101). Let-7e was found to be capable to prevent PCT progression by directly inhibiting the translation of *HMGB1* mRNA (102).

Other miRNAs found to be downregulated in PTC but with unknown molecular mechanism are miR-140-3p, miR-99a, miR-374a, miR-372, miR-363, miR-299-3p, miR-135b, miR-107, miR-103, miR-122-5p, and miR-10a-5p (22, 54, 55, 103). It is notable that miR-122-5p is expressed in both malignant and benign thyroid tumors in comparison to adjacent normal tissue (103). MiR-654-3p, miR-361-5p (via *ROCK1*), miR-497 (via *Akt3*), miR-744 (via *NOB1*), miR-613 (via *SphK2*), miR-4500 (via *PLXNC1*), miR-577 (via *SphK2*), miR-29a-3p (via *OTUB2*), miR-101 (via *RAC1*), miR-195 (via *CCND1* and *FGF2*), miR-329 (via *WNT1*), miR-4728 (via *SOS1* and MAPK signaling pathway), miR-199a-5p (via *SNAI1*), miR-758-3p (via *TAB1*), miR-219-5p (via *ERα*), miR-206 (via *MAP4K3*), miR-128 (via *SphK1*), and miR143-3p (via *MSI2*) were found in lower levels in PTC specimens and cells with enhanced cell proliferation, migration, and invasion (104–120) (**Table 2**). The effects of upregulated miRNA in papillary thyroid cancer cell lines and tissue are summarized in **Supplementary Table 2**.

**Table 2:** Downregulated miRNAs in papillary thyroid cancer.

MiRNA	Tissue	Plasma/Serum
7	+	
791	+	
144	+	
1266	+	
335	+	
718	+	
148a	+	
9-5p	+	
449	+	
202-3p	+	
126	+	
622	+	
150	+	
205	+	
204-5p	+	
7-2	+	
DICER	+	
451a	+	
215	+	
766	+	
431	+	
486-5p	+	
940	+	
16	+	
199a-3p	+	+
26a-5p	+	
564	+	
15a	+	
30c-2-3p	+	
876-5p	+	
138-5p	+	
139	+	
139-3p	+	
139-5p	+	
138-1-3p	+	
504	+	
152	+	
873	+	
199-5p	+	
20b	+	
326	+	
TG	+	
369-3p	+	
448	+	
Let-7a	+	
Let-7b-5p	+	+
Let-7e	+	
Let-d	+	
Let-7f	+	
Let-g	+	
140-3p	+	+
99a	+	
374a	+	
372	+	
363	+	
299-3p	+	
135b	+	
107	+	
103	+	
122-5p	+	
654-3p	+	
361-5p	+	
497	+	
744	+	
613	+	
4500	+	
577	+	
29a-3p	+	
101	+	
195	+	
329	+	
4728	+	
199	+	
758-3p	+	
219-5p	+	
206	+	
128	+	
143-3-p	+	
10a-5p	+	
150-5p		+
146a-5p		+
342-3p		+
190a-5p		+
95-3p		+
5010-3p		+

### MicroRNAs Deregulated in PTC Serum

A remarkable amount of genetic studies reported deregulated miRNAs in patients’ serum and plasma, indicating their potential role as valuable biomarkers for PTC development, metastasis, and recurrence risk, with great sensitivity and specificity (121). MiR-25-3p, -451a, -146b, -30a-5p, -106a, -155, and let-7e upregulation in serum and plasma in PTC account for an important tool for the diagnosis of PTC with sensitivity and specificity of more than 68% (122–125). MiR-190 and -95 could also be useful biomarkers for malignancy, since both of them have been found overexpressed in serum (126). According to the Graham et al. study, serum miRNAs 146a-5p and -199-3p were found to be downregulated and miR-10a-5p and let-7b-5p upregulated in PTC versus benign thyroid pathologies, while miR-150-5p, -146a-5p, and 342-3p were downregulated and miR-191-5p, -93-5p, and let-7b-5p were overexpressed in PTC versus normal thyroid (127). The next-generation sequencing was applied in order to compare the expression of PTC exosomes with nodular goiter (NG). MiR-598-5p, miR-3161, miR-4644, miR-6516-5p, and miR-1283 were found only in the PTC serum. However, mir-5189-3p, found to be highly expressed in the PTC serum, was characterized as the optimum biomarker for the distinction between PTC and NG. On the other hand, the concentration of mir-5010-3p was significantly low detected in the serum of PTC patients (128). Using small RNA sequencing, Dai et al. found that miR-376a-3p, miR-4306, miR-4433a-5p, and miR-485-3p were significantly upregulated in patients’ serum with PTC compared to samples from patients with benign thyroid nodules or healthy control (129). However, the authors suggest only miR-485-3p and miR-4433a-5p as putative biomarkers due to their diagnostic accuracy, whereas overexpression of 485-3p is indicative for higher risk. In another study, miR-221-3p, -146a-5p, -222-3p, 24-3p, 146b-5p, -191-5p, 103a-3p, and -28-3p were found in high levels in patient’s serum prior to thyroidectomy compared with control samples, suggesting them as possible biomarkers. On the other hand, the serum levels of miR-95-3p and -190a-5p were very low, almost undetectable (130). It is of great importance that post-operatively the miR levels were found lower, even more significant for miR-221-3p and 146a-5p (128). MiR-222 was found to be increased in the serum of patients with PTC who carried the BRAF V600E mutation. MiR-181a and -146a differ between cancerous and benign lesions and pre- and post-operatively (131). Similar results were reported by Yorurker et al. who demonstrated that the levels of miR-21, -151-5p, -221, -222, and -31 in the serum were indicative of TNM stage, metastasis, and tumor size. After thyroidectomy, miR-222, -221, and -146b levels were decreased and associated with advanced tumor stage, tumor size, and recurrence (132, 133). MiR-22 was found to be upregulated in both the tissue and serum of PTC patients and was believed to be a potential biomarker for metastatic disease (134). The exosomal miR-423-5p was found to be elevated in the serum of patients with PTC according to the study of Ye et al. (135).

The differential diagnosis between follicular and PTC versus benign tumors could be based on changes in plasma miRNAs. MiR-31 was found to be upregulated in PTC, while miR-21 was exceeded in follicular TC. MiR-181a was inversely expressed in both PTC and FTC and could find application as a helpful biomarker to distinguish them (121). The discrimination between benign and malignant thyroid tumors was assisted by the detection of miR-221, -222, -146b, and -21 in the serum, which were increased in PTC and reduced after surgery. Nevertheless, the increased serum levels postoperative was found to correlate with poor prognosis (136, 137) (**Tables 1**, **2**).

### MicroRNAs Detected in FNA

Fine-needle aspiration (FNA) is widely established as an ultrasound-guided minimal invasive method, useful for the differential diagnosis between benign and malignant thyroid lesions. As miRNAs take the forefront in diagnostic process, their aberrant expression was also examined in the FNA specimen in order to contribute to accurate diagnosis. All aforementioned deregulated miRNAs could be also examined in FNA. The molecules already demonstrated in international literature will be mentioned subsequently. MiR-30a-5p may be a diagnostic marker in FNA tissues, as it was found to be elevated also in the serum (123). MiR-222, -214, and -181b were found upregulated in FNA samples in cases suspected for PTC and associated with tumor aggressiveness (138). MiR-146b, -31, -551b, -221, and -375 were reported to give accurate results in FNA samples tested for PTC vs. FTC vs. Hurtle carcinoma vs. benign goiter (139) (**Table 3**).

**Table 3:** MiRNAs detected in FNA samples.

MiRNA	FNA
30a-5p	+
222	+
214	+
181b	+
146b	+
31	+
551b	+
221	+
375	+

### Debatable Deregulated MicroRNAs in PTC

In the international literature, there are a few significant differences among the studies regarding miRNA deregulation in PTC development.

Let -7d,-7g, -7e, and -7f miRNAs were reported to be deregulated in PTC cell lines. Let- 7e and -7g were found to be positively regulated according to Liu et al., while Perdas et al. demonstrated different regulatory directions (let -7f downregulated in TPC cell line, -7d and 7b upregulated in TPC and IHH4 cell line, respectively) (53, 54). By targeting the cell cycle, miR-214 downregulation caused PSMD10 overexpression, which through metallopeptidase MMP2 and MMP9 action and GSK−3β/β−catenin and AKT signaling accelerated cell proliferation and migration. Furthermore, miR-214 is associated with lymph node metastasis, tumor size, and advanced TNM stage (140). Contrariwise, Zarkesh et al. reported that miR-214 is elevated in PTC tissue (138). MiR-30a is downregulated according to Morani et al. and upregulated according to Peng et al. (103, 141). MiR-15a was found to be negatively regulated, acting *via* the Akt pathway, and associated with bilateral tumor, multicentricity, extrathyroidal extension, metastatic disease, and advanced TNM stage, while it was included in the upregulated group of miRNAs by Liu et al. (54, 89, 142). MiR-524-5p targeted *FOXE1* and *ITGA3* gene expression and was found to be involved in cell autophagy process and demonstrated as inconsistently regulated by two different studies (54, 143). MiR-31, negatively regulated, was described to target *HuR* gene expression and to induce malignant progression of PTC, while Suresh et al. reported miR-31 as an elevated molecule in the PTC specimen (38, 144). MiR-375 downregulation was found to enhance *ERBB2* gene expression according to Wang et al.’s study, whereas it was positively regulated in PTC according to Saiselet et al.’s study (94, 145). MiR-509 inhibited the *PAX6* gene expression and acted as a tumor suppressor, reported as downregulated in PTC by Zhang et al., while it was found to be upregulated in another study (94, 146). MiR-98 was found to be positively expressed in Liu et al.’s study and negatively expressed in Suresh et al.’s study (38, 54). MiR-137 was found to be downregulated, affecting *CXCL12* gene expression and was associated with TNM stage and nodal metastasis, whereas Zarkesh et al. described it to be overexpressed in PTC tissues (138, 147).

Furthermore, many polymorphisms have been detected in miRNAs, increasing the risk for PTC development. MiR-146a, let-7 and miR-181 polymorphisms are correlated with higher risk for PTC (148, 149). Let-7 rs10877887 polymorphism seems to increase the risk for PTC, and rs13293512 is related to advanced risk for lymph metastasis (150). MiR-608 rs4919510, miR-149 rs2292832, and miR-34b/c rs4938723 polymorphisms were found to be associated with susceptibility for PTC progression (151–153).

Let-7e, miR-181b, -135a, -15b, -320, and -484 were described to be related to familial PTC (154).

### Other Noncoding RNAs Deregulated in PTC—LncRNA and CircRNA

As genome-sequencing methods developed, new molecules have been discovered preliminarily. Long noncoding RNAs (lncRNAs) are a large (with a length of more than 200 nucleotides) and diverse group of transcribed RNA molecules that do not encode proteins. They account for the major part of the noncoding transcriptome and are important regulators of gene expression and have a wide range of functions in cellular and developmental processes (155). Circular RNA (circRNA) consists a class of one-stripe RNA whose 3’ and 5’ ends are covalently linked. These are produced by the alternative splicing, a process during which different proteins are encoded by one single gene, when different exons may be included or not in the final mRNA (156).

Regarding PTC, there are a few studies published about lncRNA and circRNA and their role on PTC development. They combine lncRNA with microRNA and related genes to create the genetic pathway responsible for PTC. Zhao et al. reported the results of their study on the regulatory network of lncRNAs and suggested several possible functional combinations of miRNAs and lncRNAs such as lncRNA AC108463.1 with miR-221, miR-222, miR-876, miR-150, and miR-205 (157). LncRNA TUG1 functions as an oncogene, as it targeted the mir-145/ZEB1 pathway and, by inhibiting *TUG1* gene expression, promoted cell proliferation, migration, and EMT formation (158). When LncRNA LINC00313 is upregulated, miR-4429 is downregulated and cell proliferation is induced in PTC, while the prognosis is worsening (158). Overexpression of LncRNA BISPR was associated with miR-21-5p, which was downregulated in PTC and both influenced cell invasiveness (159). LncRNA RMRP collaborated with miR-675 and was found to be downregulated in PTC cell lines, targeting *MAPK1* gene expression, and was found to be related to advanced TNM stage and lymph node metastasis (160). LncRNA UCA1 and LINC00514 were found to be overexpressed in PTC cells and acted as an endogenous RNA competitor for miR-204 and promoted PTC (161, 162). Lnc PTCSC3, a member of the Wnt/β-catenin pathway, was found to be downregulated sponging miR-574-5p (163). Another molecular pathway, PI3K/Akt is involved in the lncRNA/miRNA axis, as lncRNA ABHD11-AS1 sponging for miR-1301-3p targeting *STAT3*, as it was found to be upregulated in PTC (164). LncRNA TTN-AS1 seems to target the same pathway PI3K/Akt through miR-153-3p/*ZNRF2* regulation (165). LncRNA HOTTIP was found to upregulate the miR-637/Akt1 axis, enhancing PTC development (166). LncRNA GAS8-AS1 was detected to be downregulated in PTC cell lines and inhibited cell proliferation. It targeted *CCND2*, mediated by miR-135-5p (167). LncRNA DGCR5 acted as another tumor suppressor. However, it was downregulated in PTC and interacted with miR-2861, which was upregulated (168). LncRNA HOXA-AS2, being upregulated in PTC cell lines and by repressing miR-520c-3p and its target gene *S100A4*, seems to promote PTC progression (169). LncRNA TNRC6C-AS1 was found to be positively regulated in PTC and by repressing miR-129-5p, suggesting another PTC development axis (170).

LncRNAs have been correlated to TNM stage, prognosis, extrathyroidal extension, lymph node, or distant metastasis of PTC. Regarding these, lncRNA NEAT1_2 overexpression, sponging mir-106b-5p and enhancing ATAD2 protein expression, plays an important role in TNM stage and tumor size (171). Through lncRNA NEAT1 upregulation and miR-129-5p inhibition, another pathway is suggested for PTC (172). miR-129 through *MAL2* gene is downregulated in PTC as proved by another study (173). LncRNA PVT1 was found to be upregulated in PTC—combined with *IGF1R* overexpression and miR-30a downregulation—and is related to TNM stage, lymph node metastasis, and tumor infiltration (174). 

Regarding circular RNAs, circRNA ZFR plays an oncogenic role in PTC by regulating the miR-1261/*C8orf4* axis (175). CircNUP214 acted as an oncogene by regulating *ZEB2* through binding to miR-145 (176). Circ0004458 was found to be associated with miR-885-5p, negatively regulated in PTC and targeting *RAC1* gene expression (177). Circ- ITCH regulated miR-22-3p, and they moderated together the PTC progression *via* the Wnt/β-catenin signaling pathway (178). Interestingly, exosomes from the PTC cancer stem cell (PTC-CSC) model were transferred beside the transcription factors SLUG and SOX2, the lncRNA MALAT1, and the linc-ROR resulting to the induction of EMT (179). Recently, Dai et al. found DOCK9-AS2, another exosomal lncRNA derived from CSC-like cells, to be upregulated in PTC and detectable in the plasma exosomes of PTC patients (180). Exosomal DOCK9 was transmitted from PTC-CSC and promoted the stemness upon the interaction with SP1, resulting to sponging miR-1972, upregulating CTNNB1, and finally activating the Wnt/β-catenin signaling pathway. In another study, Li et al. found that estrogen receptor β (ERβ) and lncRNA *H19* are overexpressed in PTC-CSC suggesting a positive regulatory interaction of both factors in order to induce and maintain the cancer stem-like features (181). Also, lncRNA LINC00311 seems to have a key role in promoting the stem-like features by regulating the miR‐330‐5p/TLR4 pathway (182). Moreover, BANCR (BRAF-activated noncoding RNA) regulates the expression of CSC markers LGR5 and EpCAM *via* the c-Raf/MEK/ERK signaling pathway (183). More studies are necessary in order to clarify the role of lncRNA in the induction and maintenance of the stem cell-like features of the PTC cells. The new insights will contribute to the development of better therapy strategies by directly inhibiting the induction of stemness.

## Conclusion

**Figure 2:**
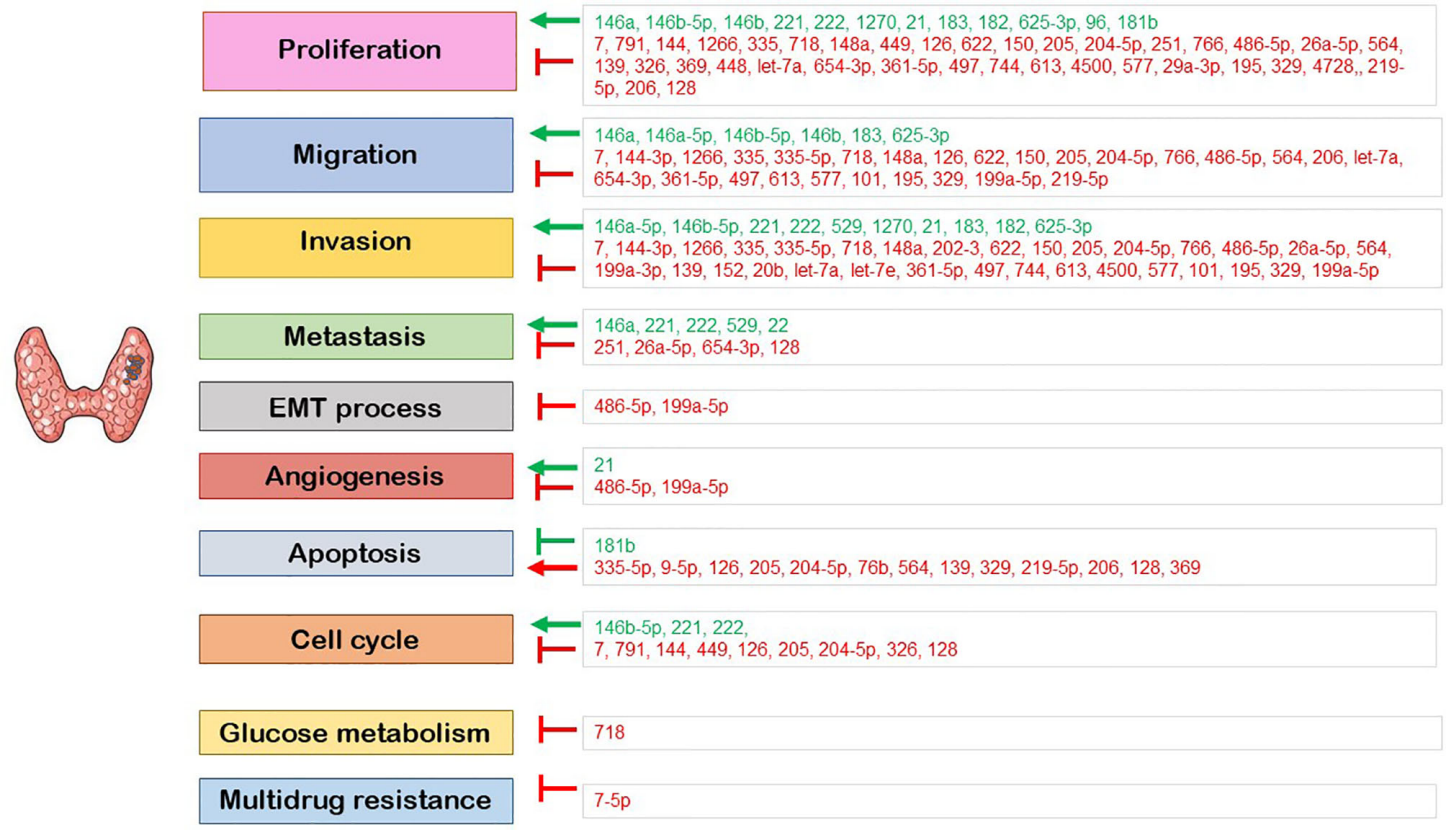
The role of the deregulated miRNA in the regulation of cellular processes. Red: downregulated miRNA; green: upregulated miRNA; ⟶: induction; ⊣: inhibition.

Deregulated miRNAs linked to PTC form a large group of molecules with complex regulatory networks, including protein coding genes, mRNAs, proteins, and regulatory enzymes (**Figure 2**). The scientific study on these molecules has not been completed yet, as many molecular pathways should be explored in the future. MiRNAs stand for potential biomarkers, useful both in diagnosis (through FNA samples and serum tests) and in treatment management of PTC, characterizing tumor stage and aggressiveness and therefore guiding the therapeutic process.

## Author Contributions

MP and AGC conducted the bibliographic search and the writing of the manuscript. SB and AC contributed to the final version of the manuscript. AC, MP, and KG provided critical feedback on the review. TP designed and supervised the review. All authors contributed to the article and approved the submitted version.

## Conflict of Interest

The authors declare that the research was conducted in the absence of any commercial or financial relationships that could be construed as a potential conflict of interest.

## Publisher’s Note

All claims expressed in this article are solely those of the authors and do not necessarily represent those of their affiliated organizations, or those of the publisher, the editors and the reviewers. Any product that may be evaluated in this article, or claim that may be made by its manufacturer, is not guaranteed or endorsed by the publisher.

## Abbreviations

AEG1, astrocyte elevated gene 1; AKT1; ARFGEF1, ADP Ribosylation Factor Guanine Nucleotide Exchange Factor 1; ATAD2, ATPase Family AAA Domain Containing 2; CCDC6, Coiled-Coil Domain Containing 6; CCND1, Cyclin D1 gene; CCND2, Cyclin G2 gene; CDK8, Cyclin Dependent Kinase 8; CDKN1C, Cyclin Dependent Kinase Inhibitor 1C; CHL1, Cell Adhesion Molecule L1 Like; CKD6, Cell division protein kinase 6; CKS2, cyclin-dependent kinase regulatory subunit 2; c-MYC; COL4A1, Collagen alpha-1(IV); CPK4, calcium-dependent protein kinase 4; CSC, Cancer Stem Cell; CXCL12, C-X-C Motif Chemokine Ligand 12; CYLD, Ubiquitin carboxyl-terminal hydrolase; EMT, epithelial–mesenchymal transition; Erα, Estrogen receptor α; Erβ, Estrogen Receptor β; ERBB2, Erb-B2 Receptor Tyrosine Kinase 2; ERBB4, Erb-B2 Receptor Tyrosine Kinase 4; FBN1, Fibrillin 1; FGF2, Fibroblast Growth Factor 2; FGFR2, Fibroblast growth factor receptor 2; FN1, Fibronectin 1; FNA, Fine Needle Aspiration; FOXE1, Forkhead Box E1; FOXO1, Forkhead Box O1; GA-binding protein; GABPA, GA-binding protein transcription factor alpha; GSK, glycogen synthase kinase; GSK-3β, glycogen synthase kinase; HMGA2, High Mobility Group AT-Hook 2; HMGB1, High Mobility Group Box 1; HOTAIR, HOX transcript antisense intergenic RNA; HuR, ELAV-like protein 1; ICAM1, Intercellular Adhesion Molecule 1; IGF1R, Insulin Like Growth Factor 1 Receptor; IGFBP5, Insulin Like Growth Factor Binding Protein 5; IRAK1, Interleukin 1 Receptor Associated Kinase 1; IRS2, Insulin receptor substrate 2; ITGA3, Integrin Subunit Alpha 3; KDM5B, Lysine specific demethylase 5B; KIAA1199, Cell Migration Inducing Hyaluronidase 1; LRP6, Low-density lipoprotein receptor−related protein 6; LSD1, Lysine-specific demethylase 1; MAL2, T-cell differentiation protein 2; MAP4K3, Mitogen-Activated Protein Kinase Kinase Kinase Kinase 3; MAPK, Mitogen-Activated protein kinase; MIF, Macrophage migration inhibitory factor; miRNA, microRNA; MMP2, Matrix Metallopeptidase 2; MMP9, Matrix Metallopeptidase 9; MSH2, DNA mismatch repair protein Msh2/MutS homolog 2; MSH3, DNA mismatch repair protein Msh3/MutS homolog 3; NF2, Neurofibromin 2; NKG2D, receptor the natural killer group 2 member D; NOB1, RNA-binding protein NOB1; OTUB2, Ubiquitin thioesterase OTUB2; PAX6, Paired box protein Pax-6; PDCD4, Programmed Cell Death Protein 4; PDPK1, 3-Phosphoinositide Dependent Protein Kinase 1; PLXNC1, Plexin C1; POLD3, DNA Polymerase Delta 3, Accessory Subunit; PPP2R2A, Protein Phosphatase 2 Regulatory Subunit Balpha; PRRX1, Paired Related Homeobox 1; PSMD10, proteasome 26S subunit non−ATPase 10; PSMD10, Proteasome 26S Subunit, Non-ATPase 10; PTC, Papillary Thyroid Cancer; PTEN, Phosphatase and tensin homolog; RAC1, Rac Family Small GTPase 1; RAC1, Ras-related C3 botulinum toxin substrate 1; RAGE, receptor for advanced glycation endproducts; RARβ, Retinoic acid receptor beta; RMPR, lncRNA mitochondrial RNA processing endoribonuclease; ROCK1, Endogenous Rho−associated protein kinase 1; ROR, Regulator of reprogramming; S100A4, S100 Calcium Binding Protein A4; SCAI, Suppressor Of Cancer Cell Invasion; SLC5A5, Solute Carrier Family 5 Member 5; SMAD4, Mothers against decapentaplegic homolog 4; SNAI1, Snail Family Transcriptional Repressor 1; SOS1, SOS Ras/Rac Guanine Nucleotide Exchange Factor 1; SOX17, SRY-Box Transcription Factor 17; SphK1, Sphingosine Kinase 1; SphK2, Sphingosine Kinase 2; TAB1, TGF-Beta Activated Kinase 1 (MAP3K7) Binding Protein 1; TGFB1, Transforming Growth Factor Beta 1; TGF-β, transforming growth factor β; TGIF1, transforming growth factor β-induced factor 1; THRβ, thyroid hormone receptor β; TIMP3, TIMP Metallopeptidase Inhibitor 3; TNFRSF12A, Tumor necrosis factor receptor superfamily member 12A; TNM, Tumor Node Metastasis; TNRRSF12A, TNF Receptor Superfamily Member 12A; TRPM3, Transient Receptor Potential Cation Channel Subfamily M Member 3; TSPAN13, Tetraspanin 13; UCA1, urothelial carcinoma−associated 1; VEGF-A, Vascular endothelial growth factor A; VHL, Von Hippel-Lindau; WNT1, Wnt Family Member 1; WWTR1, WW Domain Containing Transcription Regulator 1; YAP1, Yes Associated Protein 1; ZEB1, Zinc Finger E-Box Binding Homeobox 1; ZEB2, Zinc Finger E-Box Binding Homeobox 2; ZNRF2, Zinc And Ring Finger 2; ZNRF3, Zinc And Ring Finger 3; ATA, American Thyroid Association

## Glossary

AEG1: astrocyte elevated gene 1
AKT1, ARFGEF1: ADP Ribosylation Factor Guanine Nucleotide Exchange Factor 1
ATA: American Thyroid Association
ATAD2: ATPase Family AAA Domain Containing 2
CCDC6: Coiled-Coil Domain Containing 6
CCND1: Cyclin D1 gene
CCND2: Cyclin G2 gene
CDK8: Cyclin Dependent Kinase 8
CDKN1C: Cyclin Dependent Kinase Inhibitor 1C
CHL1: Cell Adhesion Molecule L1 Like
CKD6: Cell division protein kinase 6
CKS2: cyclin-dependent kinase regulatory subunit 2
c-MYC: COL4A1, Collagen alpha-1(IV)
CPK4: calcium-dependent protein kinase 4
CSC: Cancer Stem Cell
CXCL12: C-X-C Motif Chemokine Ligand 12
CYLD: Ubiquitin carboxyl-terminal hydrolase
EMT: epithelial–mesenchymal transition
Erα: Estrogen receptor α
Erβ: Estrogen Receptor β
ERBB2: Erb-B2 Receptor Tyrosine Kinase 2
ERBB4: Erb-B2 Receptor Tyrosine Kinase 4
FBN1: Fibrillin 1
FGF2: Fibroblast Growth Factor 2
FGFR2: Fibroblast growth factor receptor 2
FN1: Fibronectin 1
FNA: Fine Needle Aspiration
FOXE1: Forkhead Box E1
FOXO1: Forkhead Box O1
GA-binding protein, GABPA: GA-binding protein transcription factor alpha
GSK: glycogen synthase kinase
GSK-3β: glycogen synthase kinase
HMGA2: High Mobility Group AT-Hook 2
HMGB1: High Mobility Group Box 1
HOTAIR: HOX transcript antisense intergenic RNA
HuR: ELAV-like protein 1
ICAM1: Intercellular Adhesion Molecule 1
IGF1R: Insulin Like Growth Factor 1 Receptor
IGFBP5: Insulin Like Growth Factor Binding Protein 5
IRAK1: Interleukin 1 Receptor Associated Kinase 1
IRS2: Insulin receptor substrate 2
ITGA3: Integrin Subunit Alpha 3
KDM5B: Lysine specific demethylase 5B
KIAA1199: Cell Migration Inducing Hyaluronidase 1
LRP6: Low-density lipoprotein receptor−related protein 6
LSD1: Lysine-specific demethylase 1
MAL2: T-cell differentiation protein 2
MAP4K3: Mitogen-Activated Protein Kinase Kinase Kinase Kinase 3
MAPK: Mitogen-Activated protein kinase
MIF: Macrophage migration inhibitory factor
miRNA: microRNA
MMP2: Matrix Metallopeptidase 2
MMP9: Matrix Metallopeptidase 9
MSH2: DNA mismatch repair protein Msh2/MutS homolog 2
MSH3: DNA mismatch repair protein Msh3/MutS homolog 3
NF2: Neurofibromin 2
NKG2D: receptor the natural killer group 2 member D
NOB1: RNA-binding protein NOB1
OTUB2: Ubiquitin thioesterase OTUB2
PAX6: Paired box protein Pax-6
PDCD4: Programmed Cell Death Protein 4
PDPK1: 3-Phosphoinositide Dependent Protein Kinase 1
PLXNC1: Plexin C1
POLD3: DNA Polymerase Delta 3, Accessory Subunit
PPP2R2A: Protein Phosphatase 2 Regulatory Subunit Balpha
PRRX1: Paired Related Homeobox 1
PSMD10: proteasome 26S subunit non−ATPase 10
PSMD10: Proteasome 26S Subunit, Non-ATPase 10
PTC: Papillary Thyroid Cancer
PTEN: Phosphatase and tensin homolog
RAC1: Rac Family Small GTPase 1
RAC1: Ras-related C3 botulinum toxin substrate 1
RAGE: receptor for advanced glycation endproducts
RARβ: Retinoic acid receptor beta
RMPR: lncRNA mitochondrial RNA processing endoribonuclease
ROCK1: Endogenous Rho−associated protein kinase 1
ROR: Regulator of reprogramming
S100A4: S100 Calcium Binding Protein A4
SCAI: Suppressor Of Cancer Cell Invasion
SLC5A5: Solute Carrier Family 5 Member 5
SMAD4: Mothers against decapentaplegic homolog 4
SNAI1: Snail Family Transcriptional Repressor 1
SOS1: SOS Ras/Rac Guanine Nucleotide Exchange Factor 1
SOX17: SRY-Box Transcription Factor 17
SphK1: Sphingosine Kinase 1
SphK2: Sphingosine Kinase 2
TAB1: TGF-Beta Activated Kinase 1 (MAP3K7) Binding Protein 1
TGFB1: Transforming Growth Factor Beta 1
TGF-β: transforming growth factor β
TGIF1: transforming growth factor β-induced factor 1
THRβ: thyroid hormone receptor β
TIMP3: TIMP Metallopeptidase Inhibitor 3
TNFRSF12A: Tumor necrosis factor receptor superfamily member 12A
TNM: Tumor Node Metastasis
TNRRSF12A: TNF Receptor Superfamily Member 12A
TRPM3: Transient Receptor Potential Cation Channel Subfamily M Member 3
TSPAN13: Tetraspanin 13
UCA1: urothelial carcinoma−associated 1
VEGF-A: Vascular endothelial growth factor A
VHL: Von Hippel-Lindau
WNT1: Wnt Family Member 1
WWTR1: WW Domain Containing Transcription Regulator 1
YAP1: Yes Associated Protein 1
ZEB1: Zinc Finger E-Box Binding Homeobox 1
ZEB2: Zinc Finger E-Box Binding Homeobox 2
ZNRF2: Zinc And Ring Finger 2
ZNRF3: Zinc And Ring Finger 3
