# Supplementary material for: MicroRNAs in Papillary Thyroid Cancer: What Is New in Diagnosis and Treatment
Source: Front Oncol. 2022 Feb 3;11:755097. doi: 10.3389/fonc.2021.755097 (PMC8851242; doi:10.3389/fonc.2021.755097)
Supplement: Supplementary file 1 [file Table_1.docx]

**Sup. Table 1.** *The effects of upregulated miRNA in papillary thyroid cancer cell lines and tissue*. PTC; papillary thyroid carcinoma and adjacent non-cancerous tissue, PTH; papillary hyperplasia BTL; benign thyroid lesions, NT; normal thyroid tissue, NG; nodular goiter, LNM; lymph node metastasis, TNM; Classification of Malignant Tumors

| **MiRNA** | **Cell line** | **Animal model** | **Tissue samples size** | **clinicopathological features** | **Biological function** | **Target**  **Signaling pathway** | **Ref.** |
| --- | --- | --- | --- | --- | --- | --- | --- |
| 146a | K1 c | male BALB/c nude mice | 45 PTC |  | - metastasis | Upregulation by LSD1 and HIFα  LSD1/miR-146a/GABPA axis | [11] |
| 146a | TPC-1 |  | 73 PTC | - metastasis  - recurrence  - poor prognosis | - proliferation  - migration | *IRAK1* inhibition | [14] |
| 146a |  |  | 128 PTC  120 NG  120 NT | - multifocality  - extra thyroidal invasion  - advanced stages  - LNM |  |  | [28] |
| 146a |  |  | 36 PTC |  |  | Association with *THRβ* downregulation | [20] |
| 146a-5p | K01 |  | 48 PTC |  |  | *RARB* inhibition | [17] |
| 146b-5p | TPC-1  BCPAP |  |  |  | - migration  - invasion | *SMAD4* inhibition 🡪impairment of TGF-β signaling | [12] |
| 146b-5p | TPC-1  BCPAP |  |  |  | - proliferation  - invasion | TGF-β1🡪EMT | [15] |
| 146b-5p | TPC-1  K1 |  | 60 PTC |  | - migration  - invasiveness  - metastasis | Wnt/β-catenin🡪EMT | [16] |
| 146b-5p | TPC-1  BCPAP | Female BALB/c-nude mice | 92 PTC | - metastasis  - advanced stages (III/IV)  -extra-thyroidal invasion | - proliferation  - migration  - invasion  - cell cycle progression | *CCDC6* nhibition | [18] |
| 146b | BCPAP  TPC-1 |  | 50 PTC |  | - migration  - proliferation | *IRAK1* inhibition | [13] |
| 146b | TPC-1 |  | 73 PTC | - metastasis  - recurrence  - poor prognosis | - proliferation  - migration | *IRAK1* inhibition | [14] |
| 146b | K1 |  | 48 PTC |  |  | *RARB* inhibition | [17] |
| 146b-3p | K1  HP10-3 BHP103SC_mice_ | male BALB/c nude mice | 72  FFPE |  | - migration  - invasion  - metastasis | NF2 suppression | [19] |
| 146b | TPC-1 |  | 153 PTC  32 BTL | - advanced stage  - LNM  - extrathyroidal invasion  - poor prognosis |  | Due to DNA Hypomethylation | [21] |
| 146b |  |  | 421 PTC  59 NT | - LNM |  |  | [24] |
| 146b-3p  146b-5p |  |  | 237 PTC | - LNM |  |  | [26] |
| 146b-3p  146b-5p |  |  | 36 PTC | - gender (female)  - cervical LNM  - multifocality  - extra thyroidal invasion  - advanced TNM (III & IV) |  |  | [28] |
| 221 |  |  | 36 PTC |  |  | Association with *THRβ* downregulation | [20] |
| 221 | BCPAP |  | 30 PTC |  | - inhibition of cell cycle regulation  - proliferation  - motility | HMGB1/RAGE/miR221/222 | [29, 30] |
| 221 | TPC-1  BCPAP | male BALB/c nude mice | 65 PTC |  | - proliferation  - invasion  - metastasis | *TIMP3* inhibition | [31] |
| 221 |  |  | 46 PTC  20 NT | - capsule invasion  - vascular invasion  - LNM |  |  | [32] |
| 221 |  |  | 40 PTC  20 NG | - advanced TNM  - capsular invasion  - LNM |  |  | [34] |
| 221 |  |  | 78 PTC | - PTC recurrence |  |  | [35] |
| 222 | BCPAP |  | 30 PTC |  | - inhibition of cell cycle regulation  - proliferation  - motility | HMGB1/RAGE/miR221/222 | [29, 30] |
| 222 |  |  | 46 PTC  20 NT | - capsule invasion  - vascular invasion  - LNM |  |  | [32] |
| 222 |  |  | 90 PTC  10 NG | - larger tumor  - capsular invasion  - vascular invasion  - LNM |  |  | [33] |
| 222 | TPC-1  K1 |  |  |  | - invasion  - metastasis | PPP2R2A/ AKT | [37] |
| 595 | TPC-1  KAT-5 |  | 48 PTC  33 NT |  | - migration  - invasion | *IL-22* regulation  *Sox17* inhibition | [39] |
| 1270 | TPC-1  K1 | athymic nude mice | 16 PTC |  | - proliferation  - migration | *SCAI* inhibition | [40] |
| 21 |  |  | 36 PTC |  |  | Association with *THRβ* downregulation | [20] |
| 21 | TPC-1 |  | 153 PTC  32 BTL | - advanced clinical stage  - lymph node metastasis  - extrathyroidal extension  - poor prognosis |  | Due to DNA Hypomethylation | [21] |
| 21 | BCPAP  KTC-1 |  | Sera from 20 PTC with metastasis |  | Hypoxia induced exosomes 🡪  Angiogenesis | *TGFB1* and *COL4A1* inhibition | [41] |
| 21 |  |  | 66 FFPE | -recurrence |  |  | [42] |
| 21 | TPC-1 |  |  |  | - proliferation  - invasion  - inhibition of apoptosis | *PDCD4* inhibition | [43] |
| 183 | TPC-1  BCPAP  K1  NPA |  | 38 PTC |  | - proliferation  - migration  - invasion  - inhibition of apoptosis | *PDCD4* inhibition | [44] |
| 182 | TPC-1  BCPAP |  | 10 PTC |  | - proliferation  - invasion | *CHL1* inhibition | [45] |
| 92 |  |  | 36 PTC | - LNM  - capsular invasion |  | *VHL* inhibition | [46] |
| 625-3p | SW579  TPC-1 |  | 20 PTC |  | - proliferation  - migration  - invasion | Enhanced AEG-1 expression 🡪 activation Wnt/β-catenin and JNK | [47] |
| 96 | TPC-1  K1 |  | 60 PTC |  | - proliferation  - inhibition of apoptosis | Inhibition of FOXO1  AKT/FOXO1/Bim | [48] |
| 181a |  |  | 36 PTC |  |  | Association with *THRβ* downregulation | [20] |
| 181b | TPC-1 |  | 10 PTC |  | - proliferation  - inhibition of apoptosis | Inhibition of *CYLD* | [49] |
| 155 |  |  | 86 PTC | - larger tumor size  - extra-thyroid invasion  - LNM  - advanced TNM stage |  |  | [50] |
| 34a |  |  | 499 PTC  58 NT | - extra-thyroid extension  - later tumor stage |  |  | [51] |
| 424 |  |  | 499 PTC  58 NT | - extra-thyroid extension  - later tumor stage |  |  | [51] |
| 200a-5p |  |  | 40 PTC  28 PTH | - negative correlation to TPO and CD56  - positive correlation to Galectin-3, CK19 & B-raf |  |  | [52] |
| 196a-5p  187  551-3p  99b  340  954  18a  506  578  381 |  |  | 421 PTC  59 NT | - LNM |  |  | [24] |
| 346 |  |  | 120 PTC  29 NG  131 NT | - up-regulation in PTC plasma exosomes |  |  | [55] |
| 22 |  |  | 150 PTC  100 BTL | - Metastatic disease  - B‑Raf - V600E mutation  - 1‑10 ng/ml Tg  ‑ FNAB and >10 ng/ml Tg‑FNAB. |  |  | [134] |
