# Supplementary material for: MicroRNAs in Papillary Thyroid Cancer: What Is New in Diagnosis and Treatment
Source: Front Oncol. 2022 Feb 3;11:755097. doi: 10.3389/fonc.2021.755097 (PMC8851242; doi:10.3389/fonc.2021.755097)
Supplement: Supplementary file 2 [file Table_2.docx]

**Sup. Table 2.** *The effects of downregulated miRNA in papillary thyroid cancer cell lines and tissue.* PTC; papillary thyroid carcinoma and adjacent non-cancerous tissue, PTH; papillary hyperplasia BTL; benign thyroid lesions, NT; normal thyroid tissue, NG; nodular goiter, LNM; lymph node metastasis, FTC; follicular thyroid cancer, TCV; tall cell variant, Classification of Malignant Tumors, ATC; anaplastic thyroid carcinomas.

| **MiRNA** | **Cell line** | **Animal model** | **Tissue sample size** | **Clinicopathological features** | **Biological function** | **Target**  **Signaling pathway** | **Ref.** |
| --- | --- | --- | --- | --- | --- | --- | --- |
| 7 | TPC-1  K1  BCPAP |  | 10 PTC | negative correlation:  - tumor size  - LNM | Inhibition of:  - proliferation  - migration  -invasion  Regulation of:  -cell cycle | - CKS2 axis (cyclin B1 (G2/mitotic-specific cyclin-B1) and cdk1 (cyclin-dependent kinase 1) | [56] |
| 7-5p | TPC-1 |  |  |  |  | Reduced Multidrug resistance (MDR) | [57] |
| 791 | TPC-1 BCPAP HTH83 |  | 80 non-metastatic PTC | - shorter postoperative survival  - positive correlation of expression with prognosis | Inhibition of:  - proliferation | Cell cycle inhibition (downregulation of Cyclin D1, CKD6 and CDK4 induction of cyclin inhibitor P21) | [58] |
| 144 | IHH4 |  | 63 PTC | - differentiate PTC with tumor sizes ≥2 cm | Inhibition of:  - proliferation | - Targeting *WWTR1*  Hippo signaling pathway | [59] |
| 144 | BCPAP TPC-1 | male nude mice | 64 PTC | - advanced T stage | Inhibition of:  - proliferation  - tumor growth Induction of G1 arrest | -Targeting *E2F8* /CCND1 axis | [60] |
| 144-3p |  |  | 84 PTC |  | Inhibition of:  - migration  - invasion | Target of *SphK1*  Targeting *FN1* | [61,62] |
| 1266 | K1  BCPAP TPC-1 |  | 38 PTC |  | Inhibition of:  - proliferation  - migration  - invasion | Targeting *FGFR2* | [63] |
| 335 | TPC-1 HTH83 K1 BCPAP |  | 59 PTC |  | Inhibition of:  - proliferation  - migration  - invasion | Targeting *ZEB2* | [64] |
| 335-5p | TPC-1 |  | 20 PTC |  | Induction of:  -apoptosis  Inhibition of  - migration  - invasion | Targeting *ICAM-1* | [65] |
| 718 | TPC-1 K1 |  | 15 PTC |  | Inhibition of:  - proliferation  - migration  - invasion  Regulation of:  - glucose metabolism | Targeting *PDPK1*  Akt-mTOR signaling pathway | [66] |
| 148a | TPC-1 |  | 6 metastatic PTC  6 controls | - LNM |  | STAT3 and PI3K/AKT signaling pathway | [67] |
| 148a | PTC-1 BCPAP K1 | severe combined immune-deficiency mice | 81 PTC |  | Inhibition of:  - proliferation  - migration  - invasiveness  Inhibition of:  - tumor growth | Repression of cyclin-dependent kinase 8 (CDK8) | [68] |
| 9-5p |  |  | 25 PTC   25 BTL |  | Induction of apoptosis | Targeting *BRAF* | [69] |
| 449 | TPC-1 K1 IHH-4 CGTH-W3 | male nude mice | 25 PTC |  | Inhibition of proliferation  cell cycle arrest | Suppression of β-catenin nuclear translocation  - Inhibition of Wnt/β-catenin signaling (c-Myc, cyclin D1, TCF-1 and LEF-1) | [70] |
| 202-3 | TPC-1  SW1736 BCPAP K1 |  | 96 PTC | - LNM | Inhibition of invasiveness | Inhibition of Wnt/β-catenin signaling | [71] |
| 126 | K1  B-CPAP  8505-C  MB-1  BHT-101 |  | 51 PTC  37 follicular PTC  13 undifferentiated thyroid carcinomas  13 LNM thyroid  21 BTL | - metastatic PTC  - LNM  - undifferentiated TC | - Inhibition of proliferation  - Cell cycle arrest in G0-G1  - Promotion apoptosis | Inhibition of *VEGF-A* expression | [72] |
| 126 |  |  | 30 PTC | - LNM  - tumor size  - TNM stage | Inhibition of:  -proliferation  - colony formations  - migration  - invasion  Promotion of:  - apoptosis  - cell cycle arrest at G1 | LRP6 🡪 Wnt/β‑catenin signaling | [73] |
| 622 | 8505C  TPC-1  SW1736 | BALB/c-nude mice | 42 PTC | - advanced TNM stage  - LNM | Inhibition of:  - proliferation  - migration  -invasion  Suppression of:  - tumor growth in vivo | Inhibition of *VEGF-A* expression | [74] |
| 150 | TPC-1 BCPAP CGTH-W3 HTH83 |  | 45 PTC | - TNM  - LNM  - poor prognosis | Inhibition of:  - proliferation  - migration  -invasion | Inhibition of *ROCK1* expression | [75] |
| 205 | K1  B-CPAP  8505C  MB-1  BHT-101 |  | 51 PTC  37 follicular PTC 13 undifferentiated TC  13 LNM TC  14 NG  7 NT | - LNM  -TNM (T3 and T4 carcinomas  - stage 3 and stage 4 | Inhibition of:  - proliferation  Induction of:  - cell cycle arrest in G0-G1  - apoptosis | Inhibition of *VEGF-A* expression | [76] |
| 205 | 8505-C BCPAP  TPC-1 |  | 132 PTC |  | Inhibition of:  - proliferation  - migration  - invasion | Suppression of *YAP1* | [77] |
| 204-5p | TCP-1 BCPAP | male BALB/c nude mice |  |  | Inhibition of:  - proliferation  - tumorigenicity  Induction of:  - cell cycle arrest  - apoptosis | Suppression of *IGFBP5* | [78] |
| 204-5p | BCPAP TPC-1 |  | 50 PTC | - extrathyroidal extension  - high T-stage  - LNM  - BRAF V600E mutation  - aggressive tall cell variant |  | promoter DNA methylation of *TRPM3* gene | [79] |
| DICER | BCPAP  TPC1  KTC1 |  | 28 PTC | - extrathyroidal extension  - angiolymphatic invasion  - multifocality  - LNM  - distant metastasis  - recurrence  - BRAF-V600E mutation |  |  | [80] |
| DICER | TPC-1 BCPAP  FRO 8505c |  | 7 NT  31 PTC  14 ATC |  | -regulatation cell proliferation  - silencing impairs thyroid cell differentiation |  | [81] |
| 451a |  |  | 19 PTC  5 NT | - tall cell variant  - advanced stage  - extrathyroidal extension |  | Suppression of *MIF, c-MYC* and *AKT1* and attenuates AKT/mTOR pathway activation | [82] |
| 215 | K1  BCPAP TPC-1  IHH4 | male BALB/c nude mice | 48 PTC | -  LNM | Inhibition of:  - proliferation  - metastasis | Suppression of EMT via the ARFGEF1/AKT/GSK-3β/Snail signaling | [83] |
| 766 | HTH83 TPC-1  BCPAP | female BALB/c nude mice | 47 PTC | - TNM stage  - LNM | Inhibition of:  - proliferation  - colony formation  - migration  - invasion  Induction of:  - apoptosis  Reduction of:  - tumor growth in vivo | Suppression of *IRS2* 🡪 phosphoinositide 3‑kinase (PI3K)/protein kinase B (Akt) pathway | [84] |
| 486-5p | TPC-1 BCPAP |  | 66 PTC  40 patients | - LNM | Inhibition of:  - migration  - invasion  - EMT process | Upregulation of E-cadherin  Downregulation of Vimentin expression  Downregulation of Gli1 expression  🡪  Suppression of Hedgehog (Hh) signaling pathway | [85] |
| 486-5p |  |  | 59 PTC |  |  | Suppression of *KIAA1199*  EMT axis | [86] |
| 486-5p |  |  | 507 PTC  59 NT | - cancer stage  - pathologic LN  - metastasis  - recurrence  - worse overall survival |  |  | [87] |
| 486-5p | K-1  TPC-1 | male BALB/C nude mice | 20 PTC |  | Inhibition of:  - proliferation  Induction of:  - apoptosis  Reduction of:  - tumor growth in vivo | Suppression of *FBN1* expression | [88] |
| 940 |  |  | 266 PTC  280 NG  300 healthy controls | - lower in bilateral tumor than in unilateral tumor  - extrallyroidal invasion  - cervical LNM or distant metastasis |  |  | [89] |
| 15a |  |  | 266 PTC  280 NG  300 healthy controls | - lower in bilateral tumor than in unilateral tumor  - lower in multicentricity than in unicentric tumor  - extrallyroidal invasion  - cervical LNM or distant metastasis |  |  | [89] |
| 16 |  |  | 266 PTC  280 NG  300 healthy controls | - lower in multicentricity than in unicentric tumor- extrallyroidal invasion  - cervical LNM or distant metastasis |  |  | [89] |
| 26a-5p | K1  BCPAP | 24 nude mice | 58 PTC | - advanced TNM stages  - LNM | Inhibition of:  - proliferation  - invasion  - metastasis | Targeting *Wnt5a* | [91] |
| 564 | TPC-1 BCPAP HTH83 |  | 47 PTC | - LNM  - TNM | Inhibition of:  - proliferation  - migration  - invasion  Induction of:  - apoptosis | Inhibition of *AEG-1*  Deactivation of PTEN/Akt pathway | [92} |
| 199a-3p | BCPAP KTC-1 | BALB/c nude mice | 136 PTC  52 NT |  | Inhibition of:  - migration  - invasion  - cell growth  Suppression of cancer development in vivo | Hypermethylation of the miR-199a-3p promoter  Targeting *RAP2a* and *DNMT3a* | [93] |
| 30c-2-3p  876  138  139-5p  138-1-3p  873  504  152  199-5p |  |  | 59 normal  495 PTC  8 LNM | - LNM | Tumorigenesis process of thyrocytes |  | [94] |
| 139 | TPC-1 HTH83  BCPAP |  | 43 PTC |  | Inhibition of:  - proliferation  - invasion  Induction of:  - apoptosis | Targeting FN1 | [95] |
| 152 |  |  | 499 PTC  58 NT | - LNM  - extra-thyroidal invasion | PTC:  - invasion  - progression |  | [51] |
| 20b | K1  TPC-1 | BALB/c nude mice | 47 PTC | - cervical LNM  - TNM staging | Inhibition of  - cell viability  - migration  -invasion | Repression of *SOS1* or *ERK2*  🡪  Inhibition of MAPK/ERK Signaling Pathway | [96] |
| 326 | TPC-1  BCPAP CGTH-W-3  HTh83 | female mice | 60 PTC |  | Inhibition of:  - proliferation  - clone formation ability  - cell cycle (G1-accumulation)  Reduction of tumorigenesis in vivo (decrease of tumor volume and weight) | Targeting *MAPK1* and *ERBB4*  Suppression of f Ki-67, MAPK1 and ERBB4  Inhibition of:  Vimentin, N-cadherin  Enhancement of E-cadherin | [97] |
| TG | K1 |  | 14 PTC  14 NT |  |  | Reduction of *MAP4K4* | [98] |
| 369-3p | TPC-1 GLAG-66 |  | 59 NT  363 PTC  100 FTC  36 TCV  14 unknown types | - lower overall survival | Inhibition of:  - proliferation  Induction of:  - apoptosis | Targeting *TSPAN13* | [99] |
| 448 | K-1  TPC-1  B-CPAP  8505C  BHT101 | male BALB/c-null nude mice | 87 PTC | - N stage  - LNM  - TNM stage | Inhibition of:  - proliferation  - tumor growth | KDM5B-mediated  Repression of *TGIF1* | [100] |
| Let-7f |  |  |  |  | - restricted cell proliferation | Suppression of *MAPK* activation | [53] |
| Let-7a | TPC-1  BCPAP |  | 57 PTC  30 NG |  | Inhibition of:  - proliferation  - migration  - invasion | Lin28 repress the biogenesis of mature let-7  Lin28A/let-7a/c-Myc pathway | [37] |
| Let-7a | TPC-1 BCPAP | male BALB/cA nude mice | 47 PTC  21 NT |  | Inhibition of:  - proliferation  - colony formation  - migration  - invasion  Suppression of tumor growth | Repression of *AKT2* | [101] |
| Let-7e | BCPAP  TPC-1 | male athymic BALB/c nude mice |  |  | Inhibition of:  - migration  - invasion  Suppression of tumor growth | Downregulation of *HMGB1* | [101] |
| 654-3p | TPC-1  BCPAP  KTC-2 | FVB transgenic mice  (*BRAF^T1799A^*) | 467 PTC |  | Inhibition of:  - proliferation  - migration  - metastasis | EMT markers  (*Zeb1, Zeb2, Snai1* and *Snai2)* | [104] |
| 361-5p | 8505C  TPC-1 SW1736 | BALB/c nude mice | 48 PTC |  | Inhibition of:  - proliferation  - colony formation  - migration  - invasion  - tumor growth *in vivo* | Repression of *ROCK1* | [105] |
| 497 | TPC-1  K1  HTH83 BCPAP |  | 43 PTC |  | Inhibition of:  - proliferation  - migration  - invasion | Repression of *AKT3*  miR‑497/AKT3 signaling pathway | [106] |
| 744 | TPC-1  BCPAP HTH83 |  | 31 PTC |  | Inhibition of:  - proliferation  - invasion | Repression of *NOB1* | [107] |
| 613 | TPC-1  BCPAP  K1 | BALB/c-nude mice | 20 PTC |  | Inhibition of:  - proliferation  - invasion  - migration  - tumor growth *in vivo* | Repression of *SphK2* | [108] |
| 4500 | HTH83  TPC-1  K1  NIM-1  B-CPAP |  | 50 PTC | - lower survival rate  - LNM  - tumor stage  - metastasis  - extrathyroidal extension  - multifocality | Inhibition of:  - proliferation  - invasion  -colony formation | Repression of *PLXNC1* | [109] |
| 577 | TPC-1 BCPAP  K1 |  | 35 PTC |  | Inhibition of:  - proliferation  - migration  - invasion | Repression of *SphK2* | [110] |
| 29a-3p | K1  TPC-1 |  | 98 PTC | - metastasis | Inhibition of:  - cell growth  - proliferation  - invasion | Targeting of  *OTUB2*  Suppression of  OTUB2/TRAF6/NF-κB | [111] |
| 101 | TPC-1  HTH83  293T |  | 16 PTC | - LNM | Inhibition of:  - migration  - invasion | Repression of *Rac1* | [112] |
| 195 | K1  TPC-1 | BALB/c nude mice | 38 PTC |  | Inhibition of:  - proliferation  - migration  - invasion  - tumor growth *in vivo* | Targeting *CCND1* and *FGF2*  Suppression of Wnt/β-catenin and MMP 13 | [113] |
| 329 | TPC-1  BCPAP | BALB⁄c nude mice | 20 PTC |  | Inhibition of:  - proliferation  - migration  - invasion  - tumor growth *in vivo* | Repression of *WNT1* | [114] |
| 4728 | TPC-1  K1 |  | 18 PTC |  | Inhibition of:  - proliferation | Repression of *SOS1*  🡪 MAPK signaling pathway | [115] |
| 199a-5p | TPC-1  K1 | Male BALB/c nude mice | 24 PTC |  | Inhibition of:  - migration  - invasion  - EMT  - tumor growth *in vivo* | Repression of *SNAI1* | [116] |
| 758-3p | TPC-1 BCPAP |  |  |  | Inhibition of:  - proliferation  - migration  Induction of apoptosis | Repression of *TAB1* | [117] |
| 219-5p | K1  W3 |  | 30 PTC | - sex (lower at female)  - tumor size  - LNM  - extrathyroidal invasion | Inhibition of:  - proliferation  - migration  Promotion of:  - apoptosis | Repression of *ERα* | [118] |
| 206 | TPC-1  TPC-1/euthyrox |  | 23 PTC | - Decreases the Euthyrox-resistance | Inhibition of:  - proliferation  Induction of:  - apoptosis  in euthyrox-resistant PTC cells | Targeting *MAP4K3*  Inhibition of *p38* and *JNK* signaling pathway | [119] |
| 128 | FTC-133  FTC-236 TPC-1  CAL-62  FRO  ARO  K1 | BALB/c nude mice | 24 PTC  6 FTC (follicular) |  | Inhibition of:  - proliferation  - metastasis  Induction of:  - apoptosis  - cell cycle arrest in G0/G1 phase | Repression of *SPHK1* | [120] |
